# Pre-Liver Transplant ROTEM™ Clot Lysis Index Is Associated with 30-Day Mortality, But Is Not a Measure for Fibrinolysis

**DOI:** 10.3390/jcm9103298

**Published:** 2020-10-14

**Authors:** Matthias Hartmann, Bogdan Craciun, Andreas Paul, Thorsten Brenner, Fuat H. Saner

**Affiliations:** 1 Klinik für Anästhesiologie und Intensivmedizin, Universitätsklinikum Essen, Universität Duisburg-Essen, 45122 Essen, Germany; bogdan.craciun@uk-essen.de (B.C.); thorsten.brenner@uk-essen.de (T.B.); 2 Klinik für Allgemein-, Viszeral- und Transplantationschirurgie, Universitätsklinikum Essen, Universität Duisburg-Essen, 45122 Essen, Germany; andreas.paul@uk-essen.de (A.P.); fuat.saner@uk-essen.de (F.H.S.)

**Keywords:** liver transplantation, ROTEM™, clot lysis index, fibrinolysis

## Abstract

Complex alterations of the coagulation system in end stage liver disease lead to an increased risk of bleeding and mortality. In the present study, we investigated; 1. the association of pre-liver transplant rotational thrombelastometry (ROTEM™) variables with bleeding as well as 30-day-mortality and 2. the underlying pathophysiology. After approval from the local ethics committee, rotational thrombelastometry variables, conventional laboratory coagulation values, MELD score (model of end-stage liver disease), red blood cell loss, blood product use, coagulation factors, underlying disease, and demographic data were retrospectively analysed. Pre-transplant thrombelastometry clot lysis index (CLI) and MELD were the only variables associated with mortality, bleeding and blood product use, respectively. Mortality was 4.2%, when CLI was <85%, and increased to 25.7% when the CLI was >95%. Multivariate analysis including CLI and MELD score identified the CLI as an independent and the best predictor of 30-day-mortality. Interestingly, the inhibition of fibrinolysis did neither affect CLI nor the association of the variable with mortality. Thus, fibrinolysis can be excluded as the reason for low CLI values. In conclusion, low CLI values measured before the beginning of liver transplantation are associated with reduced bleeding and mortality, but do not indicate fibrinolysis.

## 1. Introduction

Although surgical techniques and experience in the liver transplantation (LTX) setting have largely increased in the last decade, the mortality is still high. Reasons for an adverse outcome are nowadays often related to the severity of the underlying liver disease, quality of the transplant, immunosuppression and concomitant diseases of the patient [1].

End stage liver disease (ESLD) leads to decreased hepatic plasma protein production and often induces a proinflammatory disease state [2]. Accordingly, reduced plasma concentrations and activities of various proteins of the coagulant, anticoagulant and fibrinolytic pathways can often be observed [3].

As the components of the three pathways are reduced, haemostasis in ESLD has been claimed a rebalanced system [4]. During minor surgery, under “unstressed” conditions, hemostasis works properly. However, during major surgery (liver transplantation), the loss and activation of components of the three pathways can induce an imbalance of coagulant, anticoagulant and fibrinolytic pathways, resulting in bleeding or thrombosis [3].

The above-mentioned disturbances of hemostasis are relevant for the outcome of patients undergoing LTX, and can be responsible for massive bleeding, as well as thrombosis and embolism. Moreover, hepatopulmonary syndrome, portopulmonary hypertension, and sinusoidal obstruction syndrome are thought to be related to hemostasis [5,6,7].

For the bed-side monitoring of hemostasis in liver transplantation, rotational thrombelastometry (ROTEM™) is often used [2,8]. The method allows the measurement of the clot firmness in a time-dependent manner, and thus permits the judgement of coagulation and fibrinolysis in whole blood samples. The clot firmness is determined by both fibrinogen concentration and platelet count; inhibition of platelet function allows the selective determination of fibrinogen concentration (FIBTEM-assay). Rotational thrombelastometry can be performed with ellagic acid (INTEM-assay) and tissue factor (EXTEM-assay) for intrinsic and extrinsic system activation (similar to PTT and INR, respectively). Moreover, an eventual fibrinolysis can be blocked to diagnose fibrinolysis (APTEM-assay).

In the present study, we investigated whether pre-transplant variables obtained by rotational thrombelastometry and conventional laboratory coagulation tests are predictors of outcome in patients undergoing LTX.

## 2. Materials and Methods

### 2.1. Patient Data

Data from 387 consecutive LTX procedures with complete data sets were investigated after approval from the local ethics committee (09-4091; 28.10.08). Patients’ age, sex, MELD (model of end-stage liver disease) score, duration of intensive care unit and hospital-stay, early allograft dysfunction, postoperative dialysis and diseases necessitating liver transplantation were recorded. Besides mortality, bed side findings (ROTEM™) and the laboratory hemostasis findings PTT (partial thromboplastin time), INR (international normalized ratio), and platelet count, as well as the infusion volume via the rapid infusion system and the autotransfusion volume obtained from the cell salvage device were registered. Red blood cell mass loss was calculated as recently described [8]. No patient was treated with tranexamic acid before the transplantation procedure and before the reported ROTEM-measurements. Overall, 92 patients were treated with tranexamic acid during the procedure, when massive hyperfibrinolysis was detectable.

### 2.2. Procedures

After the introduction of anaesthesia with thiopental, isoflurane and fentanyl were used for the maintenance of anaesthesia. Rocuronium was used for the facilitation of endotracheal intubation. A radial artery catheter, a central venous catheter, a pulmonary artery catheter as well as transoesophageal echocardiography were used for hemodynamic monitoring. For the monitoring of eventual vena cava stenosis, a catheter was inserted in the femoral vein. A rapid infusion device (FMS-2000, Belmont Instruments Corporation, Billerica, MA, USA) was used for the treatment of hypovolemia and anaemia. In all patients without cancer, intraoperative cell-salvage was used. Surgery was performed with a vena cava replacement technique without the use of a venovenous bypass. Hemostasis was evaluated with both rotational thrombelastometry (ROTEM™ Roteg 05 device) and conventional laboratory values. Citrated whole blood samples were investigated with 4 assays: 1. EXTEM test (extrinsic system activation with tissue factor), 2. INTEM test (intrinsic system activation with ellagic acid), 3. APTEM test (tissue factor activation combined with aprotinin to inhibit fibrinolysis), and 4. FIBTEM test (tissue factor activation in presence of cytochalasin D to inhibit platelet function). Measurements of hemostasis were routinely performed before the beginning of surgery. The ROTEM values evaluated were clotting time (CT), clot formation time (CFT), angle alpha, maximum clot firmness (MCF), and the 60-min clot lysis index (CLI). 

The CLI was automatically determined by the ROTEM-device and describes the clot firmness 60 min after onset of coagulation in relation to the maximum clot firmness (in percent). Disturbances of hemostasis were treated with fibrinogen, prothrombin complex concentrates, platelets, and tranexamic acid according to the ROTEM™ results. Fresh frozen plasma was not used in our series.

### 2.3. Statistics

SPSS Statistics (version 24, IBM, Armonk, NY, USA) was used for the evaluation of data. To determine the potential association of hemostasis variables with 30-day mortality, both univariate and multivariate analyses were used. For univariate analyses, the Wilcoxon test, Kaplan–Meier curves and log rank test, univariate binary logistic regression analyses (inclusion method), as well as linear correlation analyses were used. Multivariate analyses were performed using binary logistic regression (inclusion method). Some graphs were created with SigmaPlot (Systat Inc., San Jose, CA, USA).

## 3. Results

### 3.1. Patients’ Characteristics, Baseline Clinical Data and Outcome

The present study includes 387 patients, 51% of which were male, median age was 51 years and median MELD-score was 22. 30-day mortality after LTX was 17%. Causes for 30-day mortality were sepsis (n = 37), bleeding (n = 9), transplant failure (7), ischemia of other organs (n = 2), cardiac (n = 6), and other reasons (n = 5). The MELD-score was higher in non-survivors. An extensive characterization of patients including the disease aetiology is shown in Table 1.

### 3.2. Pre-Liver Transplant Hemostasis Variables in Survivors and Non-Survivors

Conventional laboratory findings demonstrated a prolongation of the median INR and the median PTT, as well as reductions of the median fibrinogen concentration, the median platelet count, and the median antithrombin activity. The above parameters were not significantly different in survivors and non-survivors (Table 2).

Among the ROTEM variables, significant differences between 30-day-survivors and non-survivors of liver transplantation were observed for clot lysis index (CLI) in the EXTEM, INTEM, and APTEM test. CLI was higher in non-survivors than in survivors. Interestingly, there was no significant difference in clot lysis index in survivors and non-survivors, when the platelet function was inhibited in the FIBTEM assay (Table 2). All other ROTEM variables showed no significant differences between survivors and non-survivors.

### 3.3. Pre-Transplant Distribution of CLI

**Figure 1:**
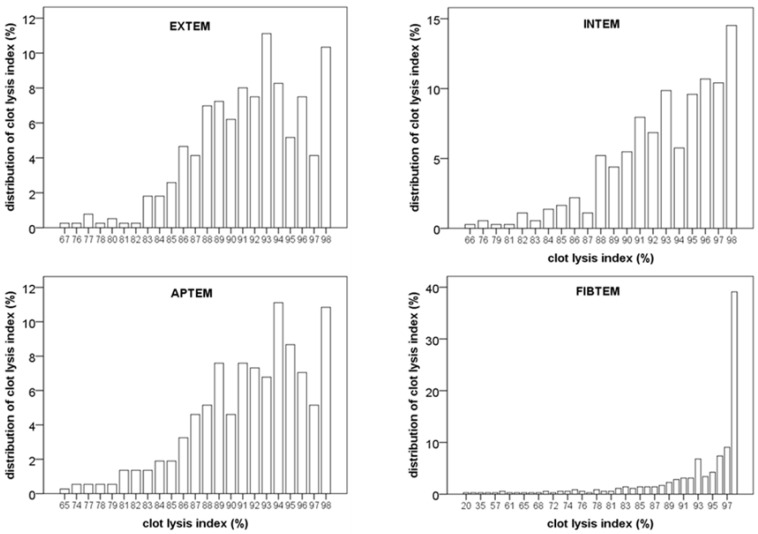
Distribution of clot lysis index at the beginning of the liver transplantation procedure. Note the broad and similar distribution in EXTEM, INTEM and APTEM. In comparison, the distribution in the FIBTEM assay was markedly smaller.

To further investigate the association of clot lysis index with mortality, the distribution of values was investigated. As shown in Figure 1, there was a broad distribution of CLI in the EXTEM, INTEM and APTEM test. Distribution of CLI was quite similar in these 3 assays. In contrast, distribution of CLI was smaller and median was higher when platelet function was inhibited in the FIBTEM test (see Figure 1 and Table 2). The platelet count was not correlated with CLI (R = –0.013; *p* = 0.823). Moreover, pre-transplant CLI was not correlated with the tranexamic acid use during the transplantation procedure (r = –0.05; r = 0.38).

### 3.4. Association of Pre-Transplant CLI and Mortality

**Figure 2:**
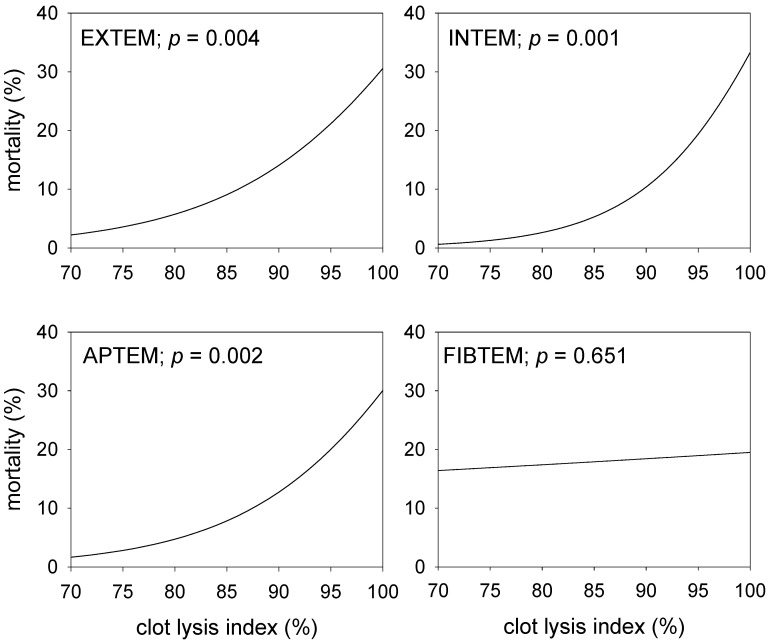
Effect of the clot lysis indices obtained with four different ROTEM™ assays before the start of the liver transplantation procedure on the 30-day mortality. Shown are calculations resulting from univariate binary logistic regression analyses and the corresponding significance levels.

To further define the association of CLI on 30-day mortality as obtained in the four thrombometric tests, univariate binary logistic regression results were used to evaluate the effects of CLI. The results, shown in Figure 2, demonstrate, that mortality significantly increases with the CLI. Notably, the results are quite similar for EXTEM, INTEM and APTEM tests, while the CLI obtained with the FIBTEM test was not associated with mortality.

### 3.5. Kaplan-Meier-Curves Dependent on Pre-Transplant CLI Categories

**Figure 3:**
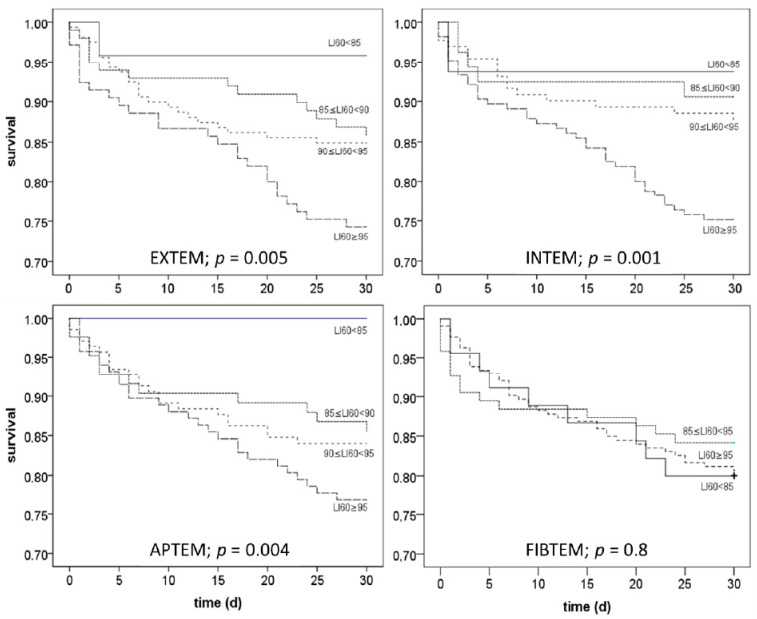
Effect of the clot lysis indices obtained with 4 different ROTEM™ assays before the start of the liver transplantation procedure on 30-day mortality. Shown are calculations resulting from univariate binary logistic regression analyses and the corresponding significance levels.

To evaluate the time course of the CLI associated mortality, CLI-values were classified (<85%, 85–90%, 90–95%, >95%) and Kaplan–Meier curves were generated for each group and the four tests used (EXTEM, INTEM, FIBTEM, APTEM). The results, shown in Figure 3, demonstrate that 30-day mortality increased with clot lysis index, when the EXTEM, INTEM, or APTEM test was used. In contrast, no differences were detectable with the FIBTEM test. 

### 3.6. Comparison of CLI and MELD as Predictors of Mortality

Multivariate analysis using binary logistic regression including CLI (as obtained with the EXTEM, INTEM or APTEM-test) and MELD demonstrated, that the CLI is the best and the only independent predictor of 30-day mortality (*p* < 0.0001).

### 3.7. Association of The Pre-Transplant CLI and Intraoperative Bleeding

**Figure 4:**
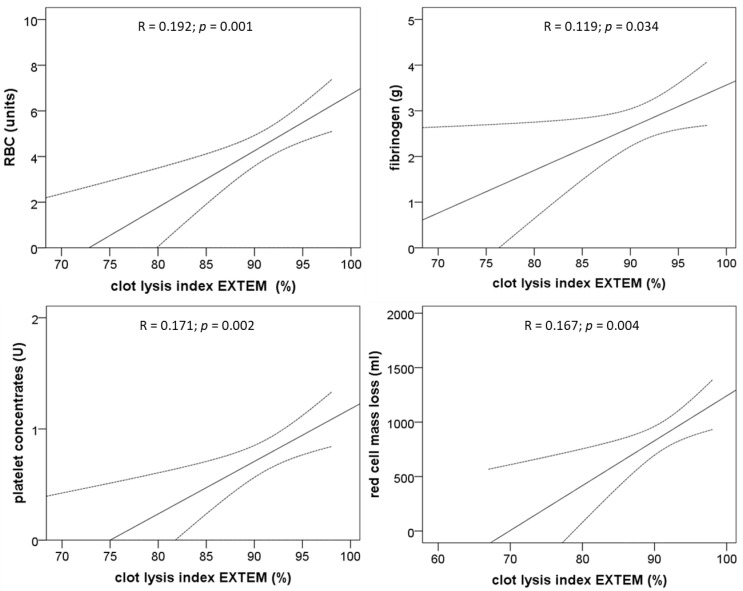
Effect of the clot lysis indices obtained with 4 different ROTEM™ assays before the start of the liver transplantation procedure on the 30-day mortality. Shown are calculations resulting from univariate binary logistic regression analyses and the corresponding significance levels.

The correlation of clot lysis index and transfusion of red blood cells, fibrinogen substitution, platelet concentrates, and loss of red blood cell mass demonstrates significant positive correlations of the CLI (EXTEM) with the above-mentioned variables (Figure 4).

### 3.8. Comparison of CLI in Absence and Presence of Aprotinin

**Figure 5:**
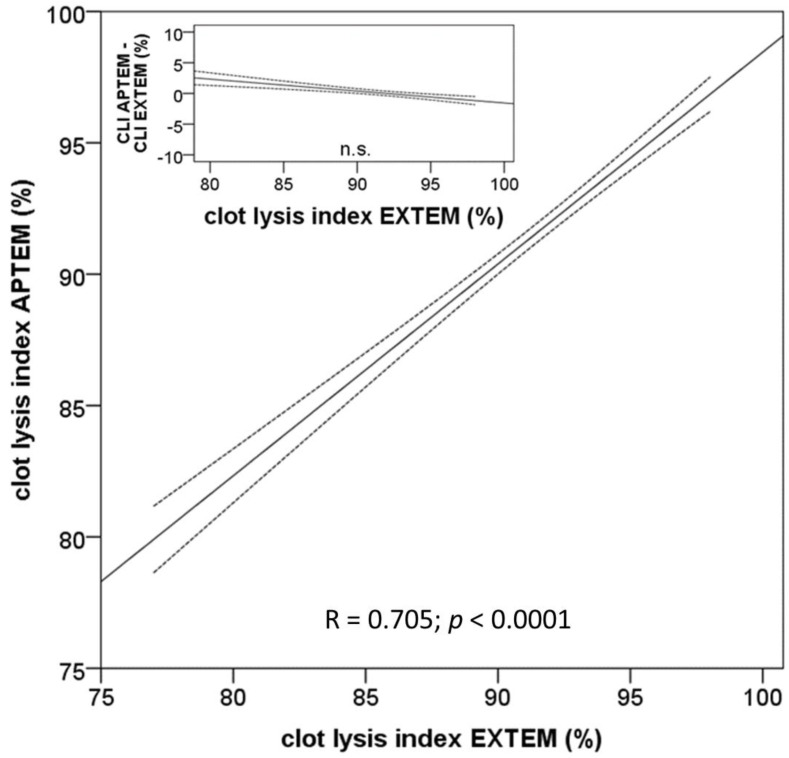
Comparison of lysis index obtained with the ROTEM™ EXTEM- and APTEM-assays before the begin of the surgical procedure. The regression curve and the confidence interval of the mean (95%) demonstrate a high correlation of both variables and thus exclude fibrinolysis in the samples. The Bland–Altman plot in the inset demonstrates that there is no significant difference between the clot lysis index obtained with the APTEM- and the EXTEM-assays.

CLI is generally used as a measure for hyperfibrinolysis. To examine whether the observed low pre-transplant CLI-values are indicative for hyperfibrinolysis, we compared the results obtained in the EXTEM- and APTEM-tests (Figure 5). In both tests, coagulation is initiated by tissue factor; the difference between both tests consists solely in the addition of aprotinin in the APTEM test to inhibit eventual fibrinolysis. Remarkably, CLI-values of both assays were highly correlated and almost identical excluding an effect of hyperfibrinolysis on the clot lysis values (Figure 5). Furthermore, the construction of a Bland–Altman plot illustrates, that there are no systemic differences between correspondent CLI values obtained with and without aprotinin (Figure 5, inset). Thus, the results regret the hypothesis, that the broad range of clot lysis index values can be explained by fibrinolysis.

## 4. Discussion

The ROTEM™ clot lysis index, measured before the start of the liver transplantation procedure, was shown to be a predictor for mortality and intraoperative bleeding. Mortality and bleeding increased with the CLI. Multivariate analysis demonstrated that the clot lysis index is a better predictor of outcome than the MELD-score. The results were highly reproducible with different tests, using either an extrinsic or intrinsic system activation with tissue factor and ellagic acid (EXTEM, INTEM), respectively. It is a further important finding, that fibrinolysis inhibition (APTEM) did neither alter the CLI nor the prediction of mortality. Thus, it must be concluded that the CLI does not serve as an indicator for fibrinolysis in the present setting. Interestingly, cytochalasin D (FIBTEM) increased the CLI and abolished its predictive value, suggesting an involvement of platelets.

### Use of Rotational Thrombelastometry in LTX

In LTX, viscoelastic assays are often used to judge the degree of hemostatic derangements and to guide the therapy with fresh frozen plasma, coagulation factors, platelets and antifibrinolytics. The most used devices for viscoelastic tests are the rotational thrombelastometry (ROTEM™) and the “classical” thrombelastography (TEG™) [9,10]. Both methods are quite similar and are capable of detecting a decrease in clot firmness with time, which is termed clot lysis index (CLI, ROTEM™) and clot lysis (TEG™), respectively. Notably, both ROTEM™ CLI (as shown in the present study) and TEG clot lysis, as demonstrated by others, are predictors of mortality [11,12]. For the comparison of TEG™ clot lysis and ROTEM™ CLI, it is important to recognize that the former variable describes the reduction of maximum clot firmness after a certain time in percent, while the latter describes the still existing clot firmness after a certain time in percent.

In contrast to the TEG-device, ROTEM™ is capable to make further conclusions on the pathophysiology of the observed hemostatic derangement, using different tests to inhibit either fibrinolysis (APTEM test) or platelet function (FIBTEM test). In the present study, it was thus possible to get important insights into the mechanisms leading to the association of CLI with mortality and bleeding. Using aprotinin (APTEM test), we excluded fibrinolysis as the cause of low CLI values, mortality and bleeding. Moreover, platelet inhibition increased the CLI, suggesting an involvement of platelets in low CLI values. The finding that the observed association of low CLI with lower blood loss and mortality is not due to fibrinolysis but is dependent on platelet function, might suggest that clot retraction is the mechanism [13,14].

Without a doubt, massive fibrinolysis may occur, especially after reperfusion of the liver transplant leading to coagulopathy, bleeding and eventual increased mortality [2]. For this reason, tranexamic acid is often used, either in a prophylactic manner or according to viscoelastic tests. Notably, many studies do not detect thrombotic complications and therefore suggest that tranexamic acid is a safe medication in this setting [8,15]. Two recent studies, however, interpreted their findings on TEG™ clot lysis with mortality as a harmful effect of hypofibrinolysis and fibrinolytic shutdown [11]. However, inhibitor experiments with antifibrinolytics and platelet inhibitors (e.g., cytochalasin D), respectively, have not been performed in these studies, and therefore, a discrimination of fibrinolysis and clot retraction was not possible. Thus, the use of aprotinin in the present study falsifies the hypothesis in literature, that “clot lysis” or “clot lysis index” as measured with viscoelastic methods at the beginning of liver transplantation procedure are indicators for fibrinolysis shutdown. Instead, the results might be compatible with differences in clot retraction. It should, however, be stated that we did not perform a classical assay for clot retraction measurement, and further examinations are therefore warranted. Of course, there are more important determinants explaining the outcome of liver transplantation, including surgical technique, transplant quality, and experience of surgeons, anaesthetists, and intensivists. Moreover, we do not show that there is a direct pathophysiologic link between CLI and outcome. Rather, it is conceivable that CLI might be a biomarker for outcome in liver transplantation. Despite these limitations, pre-transplant CLI was demonstrated to be a predictor of outcome, and the results are confirmed by others using the TEG-device [12].

## 5. Conclusions

The pre-liver transplant CLI is associated with mortality; the variable was a better predictor than the MELD score. Inhibitor experiments demonstrated that differences in CLI cannot be explained by differences in fibrinolysis. The inhibition of platelets abolished the predictive value of CLI. Probably, differences in CLI can be explained by differences in clot retraction.

## Figures and Tables

**Table 1:** Demographic and baseline clinical data of liver transplantation patients (all patients, survivors and non-survivors). Age, model of end-stage liver disease (MELD), warm and cold ischemia time, ICU-stay, ventilation time, and hospital stay as well as blood products and retransfused cell saver blood are given as median and quartiles. For statistical evaluation, the Wilcoxon test and the Chi-square test were used, as appropriate. n.s.-not significant.

	All Patients	Survivors	Non-Survivors
Patients	387	321	66
Age (years)	51.2 (0.4; 74.1)	51.0 (0.4; 69.1)	54 (0.4; 74.1) (n.s.)
Sex (m/f)	199/188	168/153	31/35 (n.s.)
MELD	22(6; 40)	22 (6; 40)	28 (7; 40) (*p* = 0.011)
Warm ischemia time (min)	34 (29; 40)	34 (29; 40)	33 (28; 42) (n.s.)
Cold ischemia time (min)	420 (343; 501)	418 (343; 494)	466 (342; 520) (n.s.)
ICU stay (days)	3.5 (2.1; 7.2)	3.5 (2.0; 7.2)	3.9 (2.4; 8.8)
Ventilation time (hours)	36 (14; 110)	30 (13; 85)	96 (30; 317)
Hospital stay (days)	24 (16; 45)	30 (13; 85)	11 (4; 27)
Early allograft dysfunction (%)	39	35	61
Post-op dialysis (%)	44	55	16
RBC (units)	3 (0; 6)	3 (0; 6)	6 (2; 10)
Fibrinogen (g)	0 (0; 4)	2 (0; 4)	3 (0; 8) (*p* = 0.02)
PCC (units)	0 (0; 1000)	0 (0; 0)	0 (0; 2000) (*p* = 0.001)
Platelets (units)	0 (0; 1)	0 (0; 1)	1 (0; 2) (*p* = 0.01)
Aetiology (cases)			
Alcohol	75	63	12
HCC	67	60	7
Hepatitis	51	48	3
Cholestatic	37	32	5
Inherited	34	26	8
Retransplant	31	17	14
NASH	19	16	3
Autoimmune	12	10	2
Toxic	11	8	3
Unknown	8	5	3
Other	42	36	6

**Table 2:** Conventional laboratory parameters and rotational thrombelastometry (ROTEM™)-variables in patients presenting for liver transplantation. Shown are median and quartiles. The Wilcoxon test was used for evaluation of differences between survivors and non-survivors.

**Conventional Laboratory Findings**				
	**All Patients**	**Survivors**	**Non-Survivors**	***p* Value**
INR	1.51 (1.29; 1.94)	1.51 (1.29; 1.93)	1.52 (1.27; 2.14)	0.519
PTT (s)	47.6 (40.5; 61.4)	47.1 (39.9; 60.6)	54.05 (41.8; 64.5)	0.144
Fibrinogen (mg/dL) mg/dL (mg/dL)	167.2 (123.1; 253.4)	167.1 (11.9; 247.0)	175.0 (123.6; 265.6)	0.641
Antithrombin (%)	44.2 (29.0; 64.2)	43.7 (29.0; 66.8)	49.3 (29.0; 63.4)	0.961
Platelets (1/nL)	85.5 (56.0; 140.8)	85.0 (56.5; 140.5)	93.0 (54.0; 158.0)	0.948
**ROTEM™ Findings**				
	**All Patients**	**Survivors**	**Non-Survivors**	***p* Value**
CT-EXTEM (s)	63.0 (48.0; 91.0)	64.0 (48.0; 92.5)	59.0 (49.5; 84.0)	0.526
CFT-EXTEM (s)	122.0 (81.0; 198.0)	122.0 (83.5; 198.5)	119.0 (79.0; 199.5)	0.742
MCF-EXTEM (mm)	51.0 (43.0; 61.0)	50.0 (43.0; 61.0)	52.5 (44.0; 63.3)	0.272
Alpha-EXTEM (^0^)	68.0 (57.0; 74.0)	68.0 (57.3; 74.0)	69.5 (56.0; 75.0)	0.716
CLI-EXTEM (%)	92.0 (89.0; 95.0)	92.0 (88.0; 94.0)	94.0 (90.0; 96.0)	0.003
CT-INTEM (s)	190.0 (164.5; 232.0)	192.0 (164.8; 234.0)	187.0 (164.0; 220.0)	0.708
CFT-INTEM (s)	113.0 (871.5; 196.5)	114.0 (73.0; 196.3)	108.0 (67.0; 202.0)	0.734
MCF-INTEM (mm)	51.0 (42.5; 60.0)	50.0 (42.0; 60.0)	54.0 (44.0; 63.0)	0.119)
Alpha-INTEM (^0^)	71.0 (61.0; 77.0)	71.0 (61.0; 77.0)	70.0 (60.0; 78.0)	0.869
CLI-INTEM (%)	94.0 (91.0; 96.5)	93.0 (90.0; 96.0)	96.0 (93.0; 98.0)	0.0001
CT-APTEM (s)	78.0 (57.0; 106.5)	76.0 (57.0; 107.0)	82.0 (59.0; 100.0)	0.908
CFT-APTEM (s)	133.0 (94.0; 212.5)	134.5 (96.3; 214.0)	131.0 (87.5; 195.5)	0.308
MCF-APTEM (mm)	51.0 (43.0; 60.5)	51.0 (43.0; 59.0)	55.0 (46.0; 64.0)	0.053
Alpha-APTEM (^0^)	65.0 (56.0; 72.0)	65.0 (56.0; 72.0)	68.5 (57.5; 73.0)	0.156
CLI-APTEM (%)	92.0 (89.0; 95.0)	92.0 (88.0; 95.0)	94.0 (90.0; 97.0)	0.003
CT-FIBTEM (s)	65.0 (48.0; 89.0)	65.0 (48.0; 89.0)	64.5 (48.5; 87.5)	0.834
CFT-FIBTEM (s)	- no calculation of the variable in more than 50% of the cases -			
MCF-FIBTEM (mm)	18.0 (11.0; 26.0)	18.0 (11.0; 26.0)	18.5 (12.0; 28.8)	0.482
Alpha-FIBTEM (^0^)	64.0 (48.5; 73.0)	63.0 (48.5; 73.0)	69.0 (47.5; 72.8)	0.807
CLI-FIBTEM (%)	96.0 (88.0; 98.0)	96.0 (87.0; 98.0)	96.0 (90.3; 98.0)	0.507
